# The New Health Regime

**Published:** 1919-07-26

**Authors:** 


### THE NEW HEALTH REGIME.

### Consultative Councils.

DETAILS are now made available of the scheme of Consultative Councils to be established in connection with the Ministry of Health.

England will have four such Consultative Councils, dealing especially with the four following matters : (*a*) Medical and allied services; (*b*) National Health Insurance (Approved Societies' Work); (*c*) Local Health Administration; (*d*) General Health Questions. In order to secure the effective working of these Councils as deliberative bodies, the membership of each Council is to be limited to a number not exceeding twenty. Each Council must include women as well as men, and the Government has undertaken that the Council on General Health Questions will consist largely of women. The members will serve for a period of three years, and no member may remain in office continuously for more than six years. Committees will be appointed for special purposes, and persons who are not members of the Councils may be added to the Committees so as to supplement the knowledge and experience possessed by the members of the Councils themselves on the matters referred to the Committees.

Regarding the principles upon which the membership of the Councils will be delivered, Dr. Addison makes it clearly understood that the object in view will not be to secure that representation should be accorded upon any Council to particular bodies or groups of bodies, or to persons resident in particular areas, but to include in each Council, so far as is practicable within the limits imposed upon its total membership, those persons, whether men or women, whose special knowledge and experience will best fit them to give advice and assistance in their collective capacity upon the matters within the province of the Council upon which they sit.

Having considered the best means of securing the application of these principles in the appointment of the Consultative Council, which will advise on Local Health Administration, Dr. Addison, as a just step, has framed a list of the bodies primarily concerned with the matters within the Council's sphere, and is inviting those bodies to suggest the names of persons suitable to become members. By this means he hopes to be in a position to select, as far as possible from names suggested, a number of persons, not exceeding sixteen or seventeen, for appointment by him as the first members of the Council.

The actual responsibility for making appointments will rest with Dr. Addison, who points out that in the discharge of this responsibility he will not be restricted to the names received from the various bodies from which he is inviting suggestions. His object in seeking nominations is to secure that he has before him as full as possible a selection of names put forward by the principal bodies concerned.

Having regard to the draft order constituting the Consultative Councils, it is clear that only a relatively small proportion of the persons whose names are suggested can in the first instance find a place upon the Councils.

It is not possible to estimate with accuracy in advance the demands which the work of the Councils is likely to make upon the time of the members, and so Dr. Addison intimates that it would not be desirable that the name of any person should be put forward for membership who would not be prepared to attend the meetings of the Councils regularly. The meetings will be held in London and may, in periods of special pressure of work, require to be held at short intervals.

Provision is made for the payment of members of the Councils, or members of Committees, to such amount as may be sanctioned by the Treasury, of subsistence allowance and reasonable compensation for loss of remunerative time and for repayment of travelling expenses.

